# Hyperforin Promotes Post-stroke Neuroangiogenesis via Astrocytic IL-6-Mediated Negative Immune Regulation in the Ischemic Brain

**DOI:** 10.3389/fncel.2019.00201

**Published:** 2019-05-07

**Authors:** Hua Yao, Yujing Zhang, Huaqing Shu, Bing Xie, Yuanfa Tao, Yin Yuan, You Shang, Shiying Yuan, Jiancheng Zhang

**Affiliations:** ^1^Department of Critical Care Medicine, Union Hospital, Tongji Medical College, Huazhong University of Science and Technology, Wuhan, China; ^2^Institute of Anesthesia and Critical Care Medicine, Union Hospital, Tongji Medical College, Huazhong University of Science and Technology, Wuhan, China; ^3^Department of Pancreatic Surgery, Renmin Hospital, Wuhan University, Wuhan, China

**Keywords:** hyperforin, IL-6, immunity, ischemic stroke, neuroangiogenesis

## Abstract

Hyperforin has been shown to be capable of promoting angiogenesis and functional recovery after ischemic stroke in our previous study. However, the exact mechanisms involved are not fully elucidated. In this study, adult male mice were subjected to 60-min transient middle cerebral artery occlusion followed by reperfusion for 28 days. Hyperforin was administrated to MCAO mice every 24 h for 2 weeks starting at 14 days post-ischemia (dpi). Then flow cytometry, quantitative Real-time PCR (RT-qPCR), western blotting, immunohistochemistry, and functional assays were performed to explore the molecular mechanisms *in vivo* and *in vitro*. Our data showed that hyperforin increased astrocytic interleukin (IL)-6 in the ischemic hemisphere via TLR4 at 28 dpi. The astrocytic IL-6 was essential to the promoting effects of hyperforin on the neural precursor cells proliferation, neuronal differentiation, angiogenesis, and functional recovery after stroke. Furthermore, hyperforin promoted the infiltration of regulatory T cells (Tregs) to the ischemic hemisphere and increased Tregs-derived cytokine IL-10 and transforming growth factor-β (TGF-β) in a manner that was dependent on astrocytic IL-6. Astrocytic IL-6 was critical to the role of hyperforin in promoting the infiltration of T-helper (Th) type 2 cells to the ischemic hemisphere and Th2-derived cytokine IL-4, relative to Th1 and Th1-derived cytokine interferon-γ (IFN-γ), which decreased during stroke recovery. After depletion of CD25^+^ Tregs, the promoting effects of hyperforin on post-stroke neurogenesis was attenuated. Moreover, blockade of IL-4 and TGF-β abrogated the promoting role of hyperforin in post-stroke neurogenesis, angiogenesis and functional recovery. Our results reveal a previously uncharacterized role of astrocytic IL-6-mediated negative immune regulation in the promoting effects of hyperforin on post-stroke neurovascular regeneration and functional recovery.

## Introduction

Under normal circumstances, the central nervous system (CNS) are isolated from the systemic immune system by the intact blood–brain barrier (BBB). The immune system could come into contact with brain antigens due to the disruption of BBB after stroke. Evidence has shown that two distinct peaks of infiltration of CD4^+^ lymphocytes to the ischemic hemisphere are observed: the first occurring shortly after stroke and the second peaking around day 14 and persisting until day 30 after stroke (Sakaguchi et al., 2009). T helper (Th) type 1 cells, Th2 cells and regulatory T cells (Tregs) are all subpopulations of CD4^+^ T cells. A Th1-type immune response is characterized by increased release of proinflammatory cytokines [interferon-γ (IFN-γ), tumor necrosis factor α (TNF-α)] that could promote the cellular immune response. A Th2-type immune response is characterized by increased release of anti-inflammatory cytokines [interleukin (IL)-4, IL-10] that could modulate the cellular immune response (Harris and Ronchese, 1999). Tregs could exert immunosuppressive and anti-inflammatory effects through secreting anti-inflammatory cytokines [IL-10, transforming growth factor-β (TGF-β)] and are highly effective in inhibiting the proliferation and cytokine release of effector T lymphocytes (Sakaguchi et al., 2009). Development of a Th1 response to myelin basic protein (MBP) is associated with worse neurological outcome 1 month after stroke (Becker et al., 2005). Whereas mucosal administration of MBP can induce a TGF-β-mediated Th2/Tregs response, contributing to a better neurological outcome for up to 1 month after stroke (Gee et al., 2007). These data indicate that the increase in proinflammatory Th1 cells could play deleterious effects on stroke outcome, whereas the increase in anti-inflammatory (Tregs and Th2) subsets of T cells could exert beneficial effects during stroke recovery. However, whether CD4^+^ T-cell subpopulations exert effects on long-term stroke outcome through modulating post-stroke neuroangiogenesis remain unclear.

Evidence has shown that hyperforin, a natural extract derived from St. John’s Wort herb commonly used in traditional medicine, has been shown to interact with the immune system (Cabrelle et al., 2008; Nosratabadi et al., 2016). Hyperforin is capable to inhibit the production of IFN-γ, with down-regulation of Th1 key transcription factor T-box and up-regulation of Th2 key transcription factor GATA-3 on IL-2/phytohemoagglutinin (PHA)-activated T lymphocytes (Cabrelle et al., 2008). Hyperforin could inhibit the differentiation of Th1 cell while promoting Tregs and Th2 cell differentiation via regulating their master transcription, and ultimately attenuate the severity of the CNS autoimmune disease (Nosratabadi et al., 2016). Our previous study has demonstrated that delayed 14-days treatment with hyperforin for 2 continuous weeks could promote angiogenesis and thus improve functional recovery after ischemic stroke (Zhang et al., 2016). However, it is unclear whether hyperforin treatment during stroke recovery could promote neuroangiogenesis through modulating CNS immunity.

Evidence from an *in vitro* study showing that IL-6 secreted from astrocytes could promote neuronal differentiation of neural stem/progenitor cells isolated from normal adult mice (Barkho et al., 2006). Furthermore, an *in vivo* study showed that IL-6 produced locally by resident brain cells could promote post-stroke angiogenesis and long-term functional recovery (Gertz et al., 2012). Our previous study found that astrocytic IL-6 is essential for enriched environment-mediated promotion of post-stroke angiogenesis and functional recovery (Chen et al., 2017). IL-6 is also critical to social support-mediated improvement in neurogenesis after ischemic stroke (Meng et al., 2015), indicating astrocytic IL-6 could promote post-stroke neuroangiogenesis. Previous study has shown that IL-6 is necessary to the antidepressant action of Hypericum perforatum extract containing 4.5% of hyperforin (Calapai et al., 2001). Antidepressant pharmacotherapy could promote post-stroke long-term functional recovery (Narushima et al., 2007; Acler et al., 2009; Jorge et al., 2010). Therefore, we propose that astrocytic IL-6 may be involved in the promoting effects of antidepressant hyperforin on neuroangiogenesis and functional recovery after ischemic stroke.

In this study, we showed that hyperforin promoted post-stroke neural precursor cells (NPCs) proliferation, neuronal differentiation, angiogenesis and functional recovery. Astrocytic IL-6 mediated the immune response toward Tregs and Th2 bias in the ischemic hemisphere and thus contributed to the promoting effects of hyperforin on neuroangiogenesis after ischemic stroke. Our findings reveal a previously unsuspected role for astrocytic IL-6-mediated negative immune regulation in the promoting effects of hyperforin on post-stroke neuroangiogenesis and long-term functional recovery.

## Materials and Methods

### Animals

Male C57BL/6J mice (8–10 weeks old, 20–25 g weight) were purchased from Vital River Laboratory Animal Technology, Co. Ltd., Beijing, China. Adult male Toll-like receptor 4 (TLR4)-deficient C3H/HeJ mice (Tlr4^Lps-d^) (Poltorak et al., 1998) and TLR4-wild-type C3H/HeN mice were purchased from Wuhan University Laboratory Animal Center. All animals, given food and water *ad libitum*, were maintained at temperature of 23 ± 11°C with 50 ± 10% relative humidity in specific pathogen-free conditions. This study was carried out in accordance with the National Institutes of Health (NIH) Guide for the Care and Use of Laboratory Animals (Publications No. 80-23) revised 1996. The protocol was approved by the Animal Care and Use Committee for experimental animals of Tongji Medical College (Wuhan, China).

### Middle Cerebral Artery Occlusion (MCAO) Model

Focal cerebral ischemia was induced by middle cerebral artery occlusion (MCAO) as previously reported (Zhang et al., 2014). Mice were anesthetized via intraperitoneal injection of 100 mg/kg ketamine and 8 mg/kg xylazine. Under an operating microscope, the right external carotid artery was incised and a 6-0 nylon monofilament with a silicone-coated round tip was inserted into the internal carotid artery until it met mild resistance for a 60 min occlusion, followed by reperfusion. A laser-Doppler probe (Periflux system 5000; Perimed AB, Stockholm, Sweden) was placed on the skull (5 mm lateral and 2 mm posterior to the bregma) to monitor the regional cerebral blood flow (rCBF) during MCAO. Mice with <25% CBF reduction following occlusion and ∼80% CBF increase upon reperfusion were included in further analyses. During the MCAO, the rectal temperature was maintained at 37 ± 0.5°C. Immediately following surgery and on a daily basis thereafter, MCAO mice in all housing groups received a subcutaneous injection of normal saline (1% v/w).

### *In vivo* Experimental Groups

Mice were divided into 22 groups randomly (Table 1): (1) Cerebral ischemia in wild-type (WT) C57BL/6 mice (MCAO); (2, 3) WT C57BL/6 mice treated with hyperforin during stroke recovery (MCAO + NS; MCAO + hyperforin): 14 days post-ischemia (dpi), a cannula was inserted to the left lateral ventricle (posterior: 0.1 mm, lateral: 0.9 mm, depth: 3 mm) using a stereotaxic instrument (RWD Life Science, Co., Ltd., Shenzhen, China) for intracerebroventricular (i.c.v.) injection. Hyperforin (0.5 μg/μl, 2 μl; Cayman Chemical, Ann Arbor, MI, United States) or normal saline (NS, 2 μl) was injected slowly (>1 min) into the left ventricle every 24 h for 2 weeks starting at 14 dpi; (4, 5) WT C57BL/6 mice treated with IL-6 neutralizing antibody (anti-IL-6) or artificial cerebrospinal fluid (aCSF) during stroke recovery (MCAO + anti-IL-6; MCAO + aCSF): mice were received i.c.v. anti-IL-6 (10 ng in 2 μl aCSF; R&D Systems, Minneapolis, MN, United States) or 2 μl aCSF every 24 h for 2 weeks starting at 14 dpi; (6, 7) WT C57BL/6 mice treated with hyperforin plus either anti-IL-6 or aCSF during stroke recovery (MCAO + hyperforin + anti-IL-6; MCAO + hyperforin + aCSF): mice were received i.c.v. either anti-IL-6 or aCSF 30 min prior to hyperforin treatment; (8, 9) WT C57BL/6 mice treated with isotype or anti-TGF-β plus anti-IL-4 (MCAO + isotype; MCAO + anti-TGF-β + anti-IL-4): mice were received i.c.v. anti-TGF-β (1 μg/μl, 1 μl; R&D System) and anti-IL-4 (0.5 μg/μl, 1 μl; R&D Systems) every 24 h for 2 weeks starting at 14 dpi; (10) WT C57BL/6 mice treated with hyperforin plus isotype or anti-TGF-β plus anti-IL-4 (MCAO + hyperforin + anti-TGF-β + anti-IL-4): mice were received i.c.v. anti-TGF-β and anti-IL-4 30 min prior to hyperforin treatment; (11-13) WT C57BL/6 mice treated with anti-CD25 or isotype (MCAO + isotype; MCAO + anti-CD25): 250 mg CD25 (Invitrogen, Carlsbad, CA, United States) was injected intraperitoneally at 14 and 21 dpi for the depletion of CD25^+^ Tregs in the ischemic hemisphere. For the control group, 250 mg isotype (Biolegend, San Diego, CA, United States) was injected intraperitoneally at 14 dpi; (14, 15) WT C57BL/6 mice treated with hyperforin plus anti-CD25 or isotype (MCAO + hyperforin + anti-CD25; MCAO + hyperforin + isotype); (16) Mouse IL-6 recombinant protein (rIL-6) was administrated to CD25-treated mice every 24 h for 7 days starting at 21 dpi. 2 μl drops of rIL-6 (R&D Systems) diluted in normal saline (0.01 μg/μl) was intranasally administrated to alternating nostrils of restrained conscious mice with a 2 min interval between applications. Drops were placed at the opening of the nostril, allowing the mice to snort each drop into the nasal cavity. A total of 10 μl of dose solution was delivered over a course of 5 min; (17, 18) Sham operation or cerebral ischemia in WT C3H/HeN mice (Tlr4^WT^ + Sham; Tlr4^WT^ + MCAO); (19, 20) Sham operation or cerebral ischemia in WT C3H/HeJ mice (Tlr4^Lps-d^ + Sham; Tlr4^Lps-d^ + MCAO); (21, 22) Cerebral ischemia in WT C3H/HeJ mice treated with normal saline or hyperforin during stroke recovery (Tlr4^Lps-d^ + MCAO + NS; Tlr4^Lps-d^ + MCAO + hyperforin).

**Table 1:** Stroke study population.

Group	Male mice	Drop-outs, *n* (reason)	Analyzed, *n* (%)	Read-out (number of mice)
MCAO	C57BL/6	2 (SAH)	15/23	RT-PCR (5)
		6 (died during or after surgery)	(65.2%)	Western blot (5)
				Immunofluorescence (5)
MCAO + NS	C57BL/6	2 (SAH)	30/40	RT-PCR (5)
		8 (died during or after surgery)	(75.0%)	Western blot (5)
				Immunofluorescence (5)
				Flow cytometry (15)
MCAO + hyperforin	C57BL/6	2 (SAH)	30/37	RT-PCR (5)
		5 (died during or after surgery)	(81.1%)	Western blot (5)
				Immunofluorescence (5)
				Flow cytometry (15)
MCAO + aCSF	C57BL/6	1 (SAH)	30/41	RT-PCR (5)
		10 (died during or after surgery)	(73.2%)	Western blot (5)
				Immunofluorescence (5)
				Flow cytometry (15)
MCAO + anti-IL-6	C57BL/6	2 (SAH)	30/40	RT-PCR (5)
		8 (died during or after surgery)	(75.0%)	Western blot (5)
				Immunofluorescence (5)
				Flow cytometry (15)
MCAO + Hyperforin + anti-IL-6	C57BL/6	1 (SAH)	30/39	RT-PCR (5)
		8 (died during or after surgery)	(76.9%)	Western blot (5)
				Immunofluorescence (5)
				Flow cytometry (15)
MCAO + Hyperforin + aCSF	C57BL/6	7 (died during or after surgery)	30/37	RT-PCR (5)
			(81.1%)	Western blot (5)
				Immunofluorescence (5)
				Flow cytometry (15)
MCAO + + isotype (i.c.v.)	C57BL/6	4 (died during or after surgery)	15/19	Western blot (5)
			(78.9%)	Immunofluorescence (5)
MCAO + + anti-TGF-β + anti-IL-4	C57BL/6	1 (SAH)	10/15	Western blot (5)
		4 (died during or after surgery)	(66.7%)	Immunofluorescence (5)
MCAO + Hyperforin + anti-TGF-β + anti-IL-4	C57BL/6	2 (died during or after surgery)	10/12	Western blot (5)
			(83.3%)	Immunofluorescence (5)
MCAO + isotype (i.p.)	C57BL/6	6 (died during or after surgery)	15/21	Immunofluorescence (5)
			(71.4%)	Flow cytometry (10)
MCAO + anti-CD25(21 dpi)	C57BL/6	2 (died during or after surgery)	5/7	Flow cytometry (5)
			(71.4%)	
MCAO + anti-CD25(28 dpi)	C57BL/6	3 (died during or after surgery)	10/13	Immunofluorescence (5)
			(76.9%)	Flow cytometry (5)
MCAO + Hyperforin + isotype (i.p.)	C57BL/6	2 (died during or after surgery)	5/7	Immunofluorescence (5)
			(71.4%)	
MCAO + Hyperforin + anti-CD25	C57BL/6	2 (died during or after surgery)	5/7	Immunofluorescence (5)
			(71.4%)	
MCAO + anti-CD25 + rIL-6	C57BL/6	1 (died during or after surgery)	5/6	Immunofluorescence (5)
			(83.3%)	
Tlr4^WT^ + Sham	WT C3H/HeN		10/10	Western blot (5)
			(100%)	Immunofluorescence (5)
Tlr4^Lps-d^ + Sham	WT C3H/HeJ		10/10	Western blot (5)
			(100%)	Immunofluorescence (5)
Tlr4^WT^ + MCAO	WT C3H/HeN	1 (SAH)	10/14	Western blot (5)
		3 (died during or after surgery)	(71.4%)	Immunofluorescence (5)
Tlr4^Lps-d^ + MCAO	WT C3H/HeJ	3 (died during or after surgery)	10/13	Western blot (5)
			(76.9%)	Immunofluorescence (5)
Tlr4^Lps-d^ + MCAO + NS	WT C3H/HeJ	3 (died during or after surgery)	10/13	Western blot (5)
			(76.9%)	Immunofluorescence (5)
Tlr4^Lps-d^ + MCAO + hyperforin	WT C3H/HeJ	2 (died during or after surgery)	10/12	Western blot (5)
			(83.3%)	Immunofluorescence (5)
	Total	101/436	335/436	
		(23.2%)	(76.8%)	


*SAH, subarachnoid hemorrhage; Sham, sham-operated; MCAO, middle cerebral artery occlusion; Tlr4^*Lps-d*^, Toll-like receptor 4 (TLR4)-deficient C3H/HeJ mice; Tlr4^*WT*^, TLR4-wild-type C3H/HeN mice; aCSF, artificial cerebrospinal fluid; NS, normal saline; rIL-6, interleukin (IL)-6 recombinant protein; anti-IL-6, IL-6 neutralizing antibody; anti-TGF-β, transforming growth factor-β neutralizing antibody; anti-IL-4, IL-4 neutralizing antibody; anti-CD25, CD25 neutralizing antibody.*

### 5-Bromo-2′-Deoxyuridine (BrdU) Labeling

Mice were injected intraperitoneally twice with S-phase marker BrdU (50 μg/g body weight in normal saline; Sigma-Aldrich; Merck KGaA, Darmstadt, Germany) with 8 h between injections at 27 dpi. Mice were anesthetized and transcardially perfused 1 day following the last injection to analyze BrdU-labeling of recently proliferated cells by immunofluorescence staining.

### *In vitro* Lipopolysaccharide (LPS) Stimulation and Treatments in Astrocyte Culture

Cells from WT C57BL/6, WT C3H/HeN or C3H/HeJ mice were dissociated from ischemic or sham-ischemic hemisphere at 28 dpi as previously described (Foo, 2013). The dissociated cells were seeded into 25 cm^2^ flasks coated with poly-L-lysine and cultured in complete medium (DMEM/F-12 + 1% penicillin-streptomycin (10,000 U/ml) + 10% FBS). The culture medium was renewed every 3 days. After 7 days of culture, non-astrocytic cells (including neurons and microglia) were detached from culture flasks by shaking at 37°C for 18 h at 180 revolutions/minute and removed by changing the culture medium. Adherent cells were harvested by trypsinization. The trypsinized cells were then resuspended in complete medium for 30 min and plated in the culture flasks (5 × 10^5^ cells/ml). The cells were purified by repeated trypsinization and seeded at a density of 5 × 10^5^ cells/ml.

Purified astrocytes were treated with LPS (100 ng/ml; Sigma, St. Louis, MO, United States) with or without the addition of hyperforin (0.8 μM, Cayman Chemical, Ann Arbor, MI, United States). At 16 h after treatment, the cells were collected for western blotting or fixed with 4% paraformaldehyde for immunofluorescence labeling. The cell culture supernatant was collected for western blotting.

### Primary Neuronal Cell Culture

Primary cultures of neurons were prepared from ischemic cerebral cortex of WT C57BL/6 mice at 28 dpi. Ischemic cerebral cortex was dissociated and incubated with 0.125% trypsin for 15 min at 37°C. After filtration through a 40 μm cell strainer and centrifugation at 700 × *g* for 5 min, the supernatant was discarded and maintained in 10% DMEM for 4 h. The cell pellet was fed with Neurobasal^TM^ Medium (Gibco BRL, Gaithersburg, MD, United States) containing 2% B27 Supplement (Gibco BRL), 1 mM L-glutamine (Gibco BRL), 1% penicillin plus streptomycin (10,000 U/ml; Solarbio, Beijing, China). These resuspended cells were cultured in poly-D-lysine (Sigma, St. Louis, MO, United States)-coated 12-well plates (3 × 10^5^ cells/ml). To reduce the number of non-neuronal cells, 10 μM cytosine arabinoside (Ara-C) was added on day 2 after plating. Medium was renewed after 24 h, and then half-renewed every 3 days. All experimental treatments were carried out on day 7 after cultures. Purified neurons were treated with LPS (100 ng/ml) with or without the addition of hyperforin (0.8 μM). At 16 h after treatment, the cells were collected for western blotting or fixed with 4% paraformaldehyde for immunofluorescence labeling.

### Functional Assays

The elevated body swing test (EBST) was performed to evaluate asymmetric motor behavior (Sun et al., 2012). The mice (*n* = 12/group) were held at the base of the tail and suspended 10 cm above the testing table at 28 dpi. A swing was recorded when mouse turned the upper body by >10° to either side in three sets of 10 trials. The number of turns to each direction (left or right) was recorded for each mouse. Results are expressed as the percentages of total number of swings made to the left.

The rotarod test was used to test the motor coordination and balance (Sun et al., 2012). Mice (*n* = 12/group) were trained daily on a rotarod rod (Med Associates Instruments) that accelerated from 4 to 40 rpm, over 120 s for 3 days before MCAO. After 3 days training, only those mice able to remain on the rod for 20 s at 40 rpm were subjected to MCAO. In the test session at 28 dpi, the average duration time the mouse managed to remain on the accelerating (4 to 40 rpm) rotarod from three consecutive trials was used for data analysis.

Motor coordination and balance was also investigated using the pole test (Gertz et al., 2012). Mice (*n* = 12/group) were placed vertically facing upward on the top of the pole wrapped with bandage gauze (9 mm diameter; 60 cm length) at 28 dpi. Both time taken to orientate the body completely downward and to reach the floor with all four paws were recorded.

### Lymphocytes Isolation and Flow Cytometry

Mice were killed and ipsilateral brain was removed and placed in ice-cold PBS. The brain was then washed in ice-cold PBS and cut into small pieces (as small as possible). The pieces were placed in 15 ml digestion solution [5% FBS (Sigma) + collagenase IV (1.75 mg/ml; Roche) + DNase I (0.5 mg/ml; Sigma)]. The resulting mixture was incubated at 37°C for 60 min with slow rotation. After the initial 60 min, the solution was vortexed intensely and passed through a 40 μm cell strainer. The remaining pieces were collected and placed into fresh digestion solution and the digestion procedure was repeated three times. Supernatants from all three digestions were combined, washed once in cold flow cytometry (FACS) buffer and separated by Percoll gradient (Sigma). Lymphocytes were harvested from the interphase of the Percoll gradient, followed by washing once and resuspending in FACS buffer.

For intracellular cytokine staining, cells obtained from dissection of ipsilateral brain were stained for cell-surface expression using optimal concentrations of anti-CD4-FITC or anti-CD25-APC antibodies followed by intracellular staining of Foxp3-PE, IL-4-PE or IFN-γ-APC using permeabilization buffer (eBioscience, San Diego, CA, United States). All flow cytometry antibodies were purchased from eBioscience (San Diego, CA, United States). Stained cells were measured in a FACSverse flow cytometer (BD Biosciences) and analyzed using FlowJo version 10 (TreeStar).

### Quantitative Real-Time PCR (RT-qPCR) Measurements

Total RNA from the post-ischemic hemisphere was isolated using TRIzol reagent (Invitrogen; Thermo Fisher Scientific, Inc., Waltham, MA, United States) according to the manufacturer’s instructions. cDNA was obtained using Taqman reverse trans-criptase (Applied Biosystems; Thermo Fisher Scientific, Inc.). TGF-β and β-actin cDNA were amplified using Power SYBR Green (Applied Biosystems; Thermo Fisher Scientific, Inc.). Two-step qPCR was performed (95°C for 15 s, 60°C for 60 s for 40 cycles) with specific primers for IL-6 (forward, 5′-GAGGATACCACTCCCAACAGACC-3′; reverse: 5′-GAGGGATATCTATCAGG GTCTTCAT-3′), IL-10 (forward, 5′–3′ and reverse, 5′–3′), TGF-β (forward, 5′-GTGTGGAGCAACATGTGGAACTCTA-3′ and reverse, 5′-TTGGTTCAGCCACTGCCGTA-3′), IL-4 (for-ward, 5′-ACCAGGAGCCATATCCAC-3′; reverse: 5′-TTGGAAGCCCTACAGACG-3′), IFN-γ (forward, 5′-TCAAGTGGCATAGATGTGGAAGA-3′ and reverse, 5′-GAGATAATCTGGCTCTGCAGGATT-3′) and β-actin (forward, 5′-AAGGCCAACCGTGAAAAGAT-3′ and reverse, 5′-GTGGTACGACCAGAGGCATAC-3′). The relative quantitation value is expressed as 2^-ΔΔCq^, where ΔCq is the difference between the mean ΔCq value of duplicate measurements of the sample and β-actin control.

### Western Blotting

The brain tissue of the ischemic hemisphere or primary cells were lysed in RIPA lysis buffer containing protease and phosphatase inhibitors (KeyGen Biotech, Nanjing, China). Proteins from cell culture supernatants were extracted as previously described (Parajuli et al., 2013). An equal amount of protein was resolved on a 10% SDS-PAGE gel (Beyotime Institute of Biotechnology, Shanghai, China) and blotted to a PVDF membrane (Millipore; Merck KGaA) using an electrophoresis apparatus (Bio-Rad Laboratories, Inc., Hercules, CA, United States). 5% non-fat milk/TBST was used to block the membranes to reduce non-specific binding. The membranes were subsequently incubated with primary antibodies against IL-6 (1:500; ABclonal Biotechnology, Co., Ltd., Wuhan, China), CD4 (1:1,000; Cell Signaling, Tech., Beverly, MA, United States), Foxp3 (1:1,000; Cell Signaling Tech., Beverly, MA, United States) and β-actin (1:2,000; Santa Cruz Biotechnology, Inc., Dallas, TX, United States) overnight at 4°C. Following extensive washing with TBST, membranes were then incubated with an appropriate peroxidase-conjugated secondary antibody (1:3,000; Proteintech Group, Inc.; Wuhan Sanying Biotechnology, Wuhan, China) for 2 h at room temperature. Following three washes with TBST, chemiluminescent signals were visualized using electrochemiluminescence western blotting detection reagents (Millipore; Merck KGaA) and bands were captured using an UVP gel documentation system (UVP, LLC, Phoenix, AZ, United States). Band intensity was quantified using Image J software (version 1.41; National Institutes of Health, Bethesda, MD, United States).

### Immunocytochemistry

Mice were anesthetized and perfused transcardially with normal saline and 4% paraformaldehyde in PBS (pH 7.4). Cultured cells were fixed for 15 min in 4% paraformaldehyde. Normal goat serum was used to block non-specific binding. Immunoassays were performed overnight at 4°C with diluted primary monoclonal antibodies (Cell Signaling Technology, Inc., Danvers, MA, United States) as follows: IL-6 (1:50), GFAP (1:50), phosphorylated-histone H3 (PH3; 1:50), Ki67 (1:100), BrdU (1:100), DCX (1:50), NeuN (1:100/), CD31 (1:50), and Foxp3 (1:50). In immunofluorescence, primary antibodies were detected with dylight 549-conjugated goat anti-rabbit or dylight 488-conjugated goat anti-mouse IgG secondary antibodies (1:200; Abbkine Scientific, Co., Ltd., Wuhan, China). Nuclei were stained with DAPI and slides were observed using a fluorescence microscope (BX51; Olympus Corporation, Tokyo, Japan). In immunohistochemistry, primary antibody binding was visualized by use of biotinylated secondary antibody, avidin-biotinylated enzyme complex (ABC) and diaminobenzidine (Zhongshan Goldenbridge Biotechnology, Beijing, China).

### Statistical Analysis

One-way analysis of variance (ANOVA) followed by a Newman–Keuls multiple comparison test was used when comparing more than two groups. Student’s *t*-test was used to compare differences between two groups. All data were presented as mean ± SD. *P* < 0.05 was considered statistically significant. Statistics were done using GraphPad Prism 5 software (GraphPad Software, Inc., LaJolla, CA, United States).

## Results

### Hyperforin Increased Astrocytes-Derived IL-6 via TLR4 During Stroke Recovery

**FIGURE 1:**
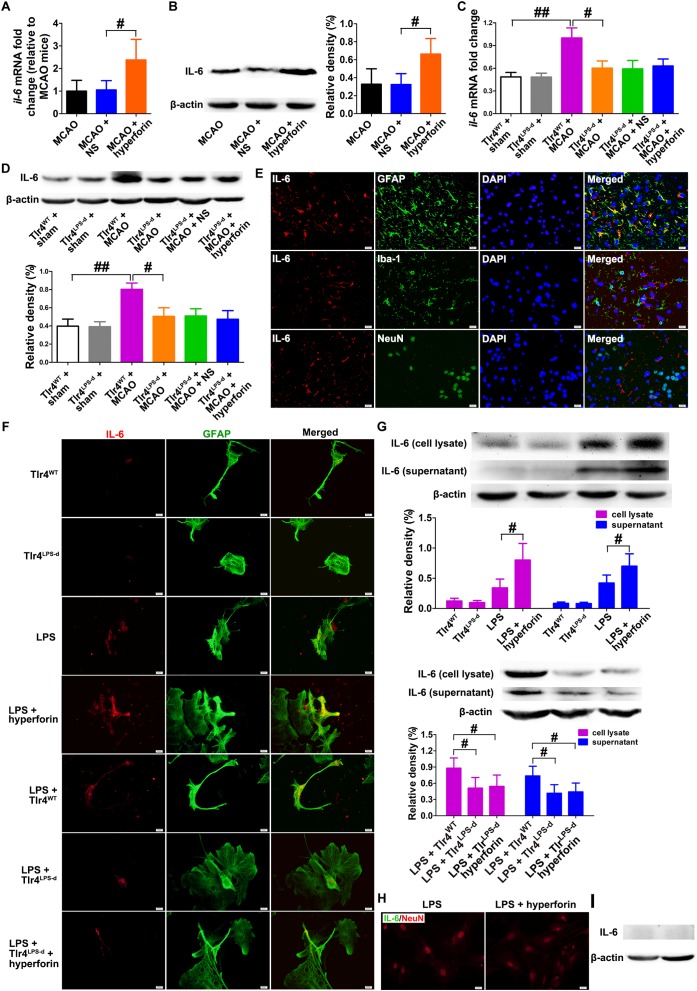
Hyperforin increased the production and secretion of IL-6 from astrocytes in a TLR4-dependent manner during stroke recovery. Levels of IL-6 in each group were measured by real-time PCR **(A)** and western blotting **(B)** in the ischemic hemisphere at 28 days post-ischemia (dpi). Data represent mean ± SD, *n* = 5; ^#^*P* < 0.05. Real-time PCR **(C)** and western blotting **(D)** analysis of IL-6 expression for each group in mice defective in TLR4 signaling (Tlr4^Lps-d^) or the corresponding wild-type mice (Tlr4^WT^) at 28 dpi. Data represent mean ± SD, *n* = 5; ^#^*P* < 0.05; ^##^*P* < 0.01. **(E)** Double immunofluorescence staining for IL-6 (red) and GFAP (green), NeuN (green), or Iba-1 (green) in the ischemic hemisphere at 28 dpi. Bar = 20 μm. **(F)** Representative fluorescence images of primary cells stained with IL-6 (red) and GFAP (green) in cultured mouse astrocytes for each group. Bar = 20 μm. **(G)** Western blotting and quantitative data for IL-6 in the culture supernatant or in the cell lysates of mouse astrocytes. Data represent mean ± SD, *n* = 5; ^#^*P* < 0.05. **(H)** Representative fluorescence images of primary cells stained with IL-6 (green) and GFAP (red) in primary cultured mouse neurons for each group. Bar = 20 μm. **(I)** Western blotting for IL-6 in the cell lysates of primary cultured mouse neurons.

In light of recent findings suggesting that hyperforin could promote angiogenesis and astrocytic IL-6 is essential to angiogenesis after ischemic stroke, we sought to investigate whether hyperforin treatment during stroke recovery could increase the IL-6 expression by astrocytes. We found that delayed 14 days treatment with hyperforin significantly increased the mRNA (*P* < 0.05; Figure 1A) and protein expression (*P* < 0.05; Figure 1B) of IL-6 in the ischemic hemisphere at 28 dpi.

TLR4 could mediate the secretion of IL-6 from astrocytes (Krasovska and Doering, 2018). Thus, we next investigated whether hyperforin increased IL-6 expression via TLR4 by using TLR4-defective C3H/HeJ mice. The mRNA (*P* < 0.01; Figure 1C) and protein expression (*P* < 0.01; Figure 1D) of IL-6 were significantly increased in TLR4-wild-type C3H/HeN mice at 28 dpi, compared to that in sham-operated TLR4-wild-type C3H/HeN mice. However, cerebral ischemia had no significantly effects on the mRNA and protein expression of IL-6 in C3H/HeJ mice (*P* > 0.05; Figure 1C,D). Moreover, compared with TLR4-wild-type C3H/HeN mice, the mRNA (*P* < 0.05; Figure 1C) and protein expression (*P* < 0.05; Figure 1D) of IL-6 in the ischemic hemisphere at 28 dpi was significantly decreased in C3H/HeJ mice. Administration of hyperforin had no significant effect on the expression of IL-6 at 28 dpi when injected into C3H/HeJ mice (*P* > 0.05; Figure 1C,D).

Given that IL-6 is critical to post-stroke angiogenesis and long-term functional recovery (Gertz et al., 2012), we performed double immunofluorescence labeling to identify the cellular source of IL-6 at 28 dpi. IL-6 IL-6 was co-expressed with glial fibrillary acidic protein (GFAP, astrocyte marker) but not with Iba-1 (microglia marker) or NeuN (neuron marker), indicating that the main cellular source of IL-6 is astrocyte during stroke recovery (Figure 1E). Furthermore, in primary astrocytes cultures from the ischemic mice at 28 dpi, IL-6 expression in both the cell lysate and culture supernatant of astrocytes treated with hyperforin was significantly increased (*P* < 0.05 and *P* < 0.05, respectively; Figure 1F,G). The expression of IL-6 in both the cell lysate and culture supernatant was significantly reduced in LPS-activated astrocytes dissociated from C3H/HeJ mice, compared to that from wild-type C3H/HeN mice (*P* < 0.05 and *P* < 0.05, respectively; Figure 1F,G). Hyperforin had no effect on IL-6 expression in the cell lysate and culture supernatant of LPS-activated astrocytes dissociated from C3H/HeJ mice (*P* > 0.05; Figure 1F,G). These data suggest that TLR4 is essential for the promoting effects of hyperforin on the production and secretion of IL-6 from astrocytes during stroke recovery.

NO expression of IL-6 protein measured by western blotting and immunofluorescence analysis was detected in LPS- or LPS plus hyperforin-treated primary cultured mouse neurons (Figure 1H,I), indicating that IL-6 upon hyperforin treatment is solely from the astrocytes and not neurons.

### Hyperforin Promoted Post-stroke Neurogenesis via IL-6

**FIGURE 2:**
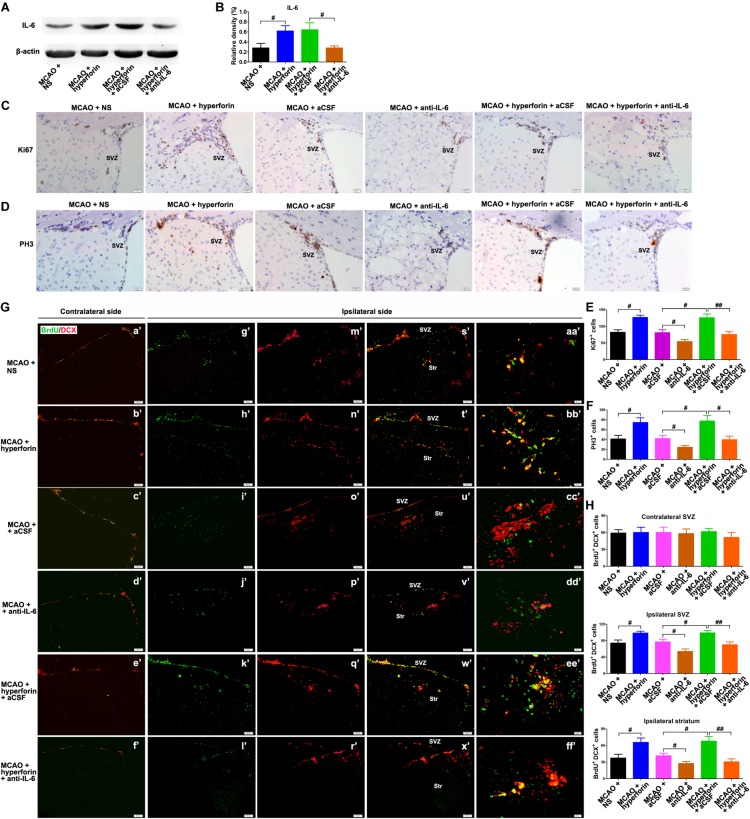
Hyperforin promoted subventricular zone (SVZ) neurogenesis via IL-6 after ischemic stroke. Western blotting images **(A)** and quantitative data **(B)** for IL-6 in the ischemic hemisphere of each group at 28 dpi. Data represent mean ± SD, *n* = 5; ^#^*P* < 0.05. Representative micrographs and quantitative determination of Ki67 **(C,E)** and phospho-histone H3 (PH3)-labeling cells **(D,F)** for each group in the ischemic SVZ at 28 dpi. Bar = 20 μm. Data represent mean ± SD, *n* = 5; ^#^*P* < 0.05; ^##^*P* < 0.01. **(G,H)** Immunofluorescence imaging and quantitative comparison of BrdU^+^ (red) cells coexpressing doublecortin (DCX) (green) for each group in the ischemic SVZ and striatum at 28 dpi. Bar = a′–x′ = 100 μm, aa′–ff′ = 20 μm. Data represent mean ± SD, *n* = 5; ^#^*P* < 0.05 and ^##^*P* < 0.01. aCSF, artificial cerebrospinal fluid.

Next, we investigated the role and mechanism of hyperforin in subventricular zone (SVZ) neurogenesis. We found that neutralizing anti-IL-6 treatment effectively decreased the protein expression of IL-6 in the hemisphere (*P* < 0.05; Figure 2A,B). The number of ki67^+^ (marker for all cell cycle phases except Go) (82 ± 8 vs. 127 ± 7, *P* < 0.05; Figure 2C,E) and PH3^+^ (marker for M-phase) cells (47 ± 7 vs. 75 ± 10, *P* < 0.05; Figure 2D,F) in the ischemic SVZ at 28 dpi was significantly increased after the treatment of hyperforin. Blockade of IL-6 with neutralizing anti-IL-6 significantly decreased the number of ki67^+^ (81 ± 9 vs. 54 ± 6, *P* < 0.05; Figure 2C,E) and PH3^+^ cells (78 ± 11 vs. 45 ± 7, *P* < 0.05; Figure 2D,F) in the ischemic SVZ at 28 dpi. Moreover, blockade of IL-6 significantly attenuated the promoting effects of hyperforin on the number of ki67^+^ (126 ± 11 vs. 75 ± 9, *P* < 0.05; Figure 2C,E) and PH3^+^ cells (78 ± 11 vs. 45 ± 7, *P* < 0.05; Figure 2D,F) in the ischemic SVZ at 28 dpi.

To further assess the cell proliferation, BrdU (S-phase marker) was administrated to mice with an 8-h interval between two injections at 27 dpi; on the following day, the mice were humanely killed by cervical dislocation to analyze BrdU incorporation into dividing cells. DCX, a neuron-specific microtubule-associated protein, is expressed by virtually all neuroblasts and functions as a marker for neuronal precursors and neurogenesis. Adult neurogenesis was detected with BrdU and DCX double marker. No significant differences in the number of BrdU^+^/DCX^+^ cells in the contralateral non-ischemic SVZ were noted among each group (Figure 2G,H). It was observed that hyperforin treatment significantly increased the number of BrdU^+^/DCX^+^ cells in the ischemic SVZ (74 ± 7 vs. 99 ± 4, *P* < 0.05) and striatum (37 ± 7 vs. 66 ± 8, *P* < 0.05) at 28 dpi (Figure 2G,H). Blockade of IL-6 significantly decreased the number of BrdU^+^/DCX^+^ cells in the ischemic SVZ (77 ± 6 vs. 54 ± 6, *P* < 0.05) and striatum (41 ± 5 vs. 27 ± 4, *P* < 0.05) at 28 dpi (Figure 2G,H). Anti-IL-6 significantly decreased the number of BrdU^+^/DCX^+^ cells in the ischemic SVZ (99 ± 6 vs. 70 ± 7, *P* < 0.01) and striatum (68 ± 9 vs. 31 ± 5, *P* < 0.01) of hyperforin-treated mice at 28 dpi (Figure 2G,H), indicating that hyperforin promotes post-stroke neurogenesis via IL-6.

### Hyperforin Promoted Post-stroke Angiogenesis and Functional Recovery via IL-6

**FIGURE 3:**
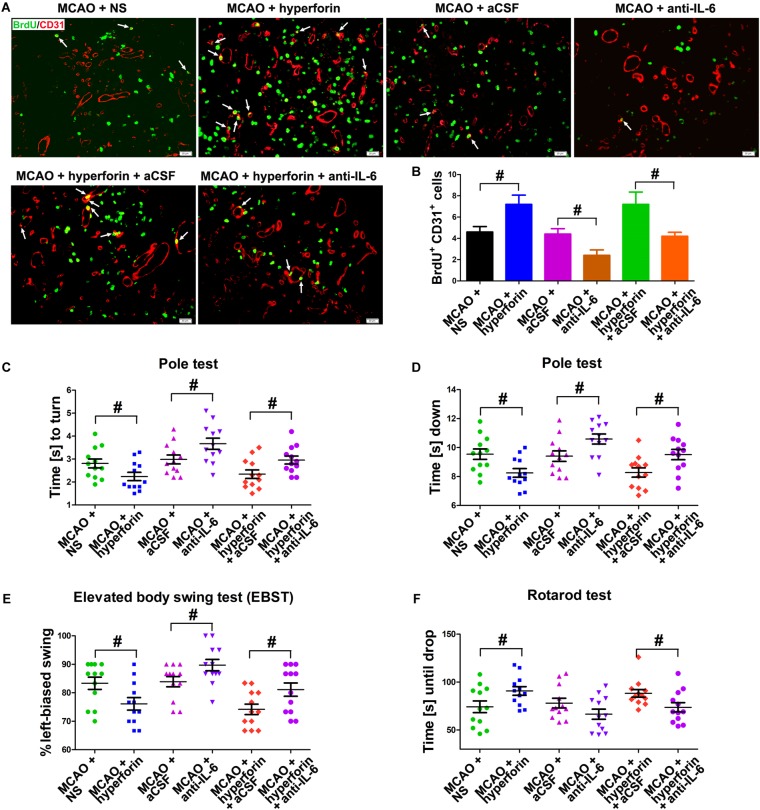
Hyperforin promoted angiogenesis and functional recovery via IL-6 during stroke recovery. **(A)** Immunofluorescence imaging for BrdU^+^ (red) cells coexpressing CD31 (green) in the ischemic hemisphere of each group at 28 dpi. Bar = 20 μm. **(B)** Quantitative comparison of BrdU^+^/CD31^+^ cells in the ischemic penumbra for each group. Data represent mean ± SD, *n* = 5; ^#^*P* < 0.05. Behavioral tests were assessed at 28 dpi, including the pole test [time to turn completely head down **(C)** and time to reach the floor **(D)**], elevated body swing test (EBST) **(E)** and rotarod test **(F)**. Data represent mean ± SD, *n* = 12; ^#^*P* < 0.05. aCSF, artificial cerebrospinal fluid.

It was previously shown that hyperforin could promote post-stroke angiogensis (Gertz et al., 2012). Here, we sought to determine whether IL-6 was involved in the promoting role of hyperforin in angiogenesis after stroke. Consistently, hyperforin treatment significantly increased the number of BrdU^+^/CD31^+^ cells in the ischemic penumbra at 28 dpi (5 ± 1 vs. 7 ± 1, *P* < 0.01; Figure 3A,B). Blockade of IL-6 significantly decreased the number of BrdU^+^/CD31^+^ cells in the ischemic penumbra at 28 dpi (4 ± 1 vs. 2 ± 1, *P* < 0.01; Figure 3A,B). The number of BrdU^+^/CD31^+^ cells in the ischemic penumbra of hyperforin-treated mice at 28 dpi was significantly decreased after anti-IL-6 intervention (7 ± 1 vs. 4 ± 1, *P* < 0.05; Figure 3A,B), indicating hyperforin increases post-stroke angiogenesis via IL-6.

Functional analysis results showed that hyperforin treatment significantly decreased the times to turn and to reach the floor on the pole test (*P* < 0.05 and *P* < 0.05, respectively; Figure 3C,D), reduced behavioral asymmetries on the EBST (*P* < 0.05; Figure 3E) and increased times until drop on the Rotarod test at 28 dpi (*P* < 0.05; Figure 3F). Blockade of IL-6 significantly increased the times to turn and to reach the floor on the pole test (*P* < 0.05 and *P* < 0.05, respectively; Figure 3C,D) and the behavioral asymmetries on the EBST (*P* < 0.05; Figure 3E). The times to turn and to reach the floor on the pole test (*P* < 0.05 and *P* < 0.05, respectively; Figure 3C,D) and the behavioral asymmetries on the EBST (*P* < 0.05; Figure 3E) were significantly increased, whereas the times until drop on the Rotarod test (*P* < 0.05; Figure 3F) at 28 dpi were significantly decreased in hyperforin-treated mice after anti-IL-6 intervention, indicating that hyperforin improves locomotor balance and coordination abilities in the delayed phases of stroke recovery via IL-6.

### Hyperforin Skewed the Immune Response to a Th2 Bias in the Ischemic Hemisphere via IL-6 During Stroke Recovery

**FIGURE 4:**
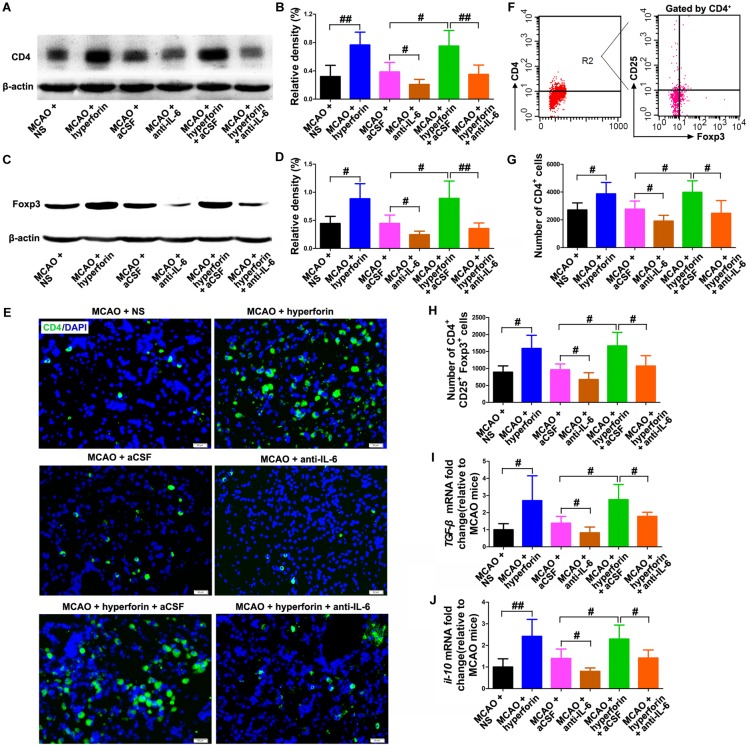
Hyperforin increased the infiltration of CD4^+^ lymphocytes and Tregs to the ischemic hemisphere via IL-6 during stroke recovery. Western blotting and quantitative data for CD4 **(A,B)** and Foxp3 **(C,D)** in the ischemic hemisphere for each group at 28 dpi. Data represent mean ± SD, *n* = 5; ^#^*P* < 0.05 and ^##^*P* < 0.01. **(E)** Representative photomicrographs of CD4 immunostaining in the ischemic hemisphere at 28 dpi. Bar = 100 μm. **(F–H)** Flow cytometry analysis of ischemic hemisphere for CD4^+^ cells and CD4^+^ CD25^+^ Foxp3^+^ regulatory T cells (Tregs) at 28 dpi. Data represent mean ± SD, *n* = 5; ^#^*P* < 0.05. Real-time PCR analysis of the mRNA expression of IL-10 **(I)** and TGF-β **(J)** in the ischemic hemisphere for each group at 28 dpi. Data represent mean ± SD, *n* = 5; ^#^*P* < 0.05 and ^##^*P* < 0.01. aCSF, artificial cerebrospinal fluid.

Evidences have shown that a second peak of infiltration of CD4^+^ lymphocytes to the ischemic hemisphere occurs around day 14 and persists until day 30 after stroke (Sakaguchi et al., 2009), and a strong accumulation and proliferation of Tregs in the ischemic hemisphere was observed for up to 30 days after stroke (Stubbe et al., 2013). Thus, we analyzed the role and mechanism of hyperforin in the infiltration of CD4^+^ lymphocytes and Tregs to the ischemic hemisphere during stroke recovery. We found that hyperforin significantly increased the protein expression of CD4 (*P* < 0.01; Figure 4A,B) and Treg-specific transcription factor Foxp3 (*P* < 0.05; Figure 4C,D) in the ischemic hemisphere at 28 dpi. CD4^+^ cells in the ischemic hemisphere at 28 dpi were markedly increased after hyperforin treatment (Figure 4E). In addition, flow cytometry results validated a promoting effects of hyperforin on the infiltration of CD4^+^ lymphocytes (2710 ± 226 vs. 3870 ± 367, *P* < 0.05) and Tregs (890 ± 84 vs. 1589 ± 173, *P* < 0.05) to the ischemic hemisphere at 28 dpi (Figure 4F–H). However, anti-IL-6 significantly decreased the protein expression of CD4 (*P* < 0.01; Figure 4A,B) and Foxp3 (*P* < 0.01; Figure 4C,D), decreased the infiltration of CD4^+^ lymphocytes (3982 ± 372 vs. 2470 ± 410, *P* < 0.05; Figure 4F,G) and Tregs (1666 ± 177 vs. 1071 ± 136, *P* < 0.05; Figure 4F,H) to the ischemic hemisphere of hyperforin-treated mice at 28 dpi. Furthermore, anti-IL-6 also abolished the promoting effects of hyperforin on the mRNA expression of Tregs-derived cytokine *il-10* and *tgf-β* in the ischemic hemisphere at 28 dpi (*P* < 0.05 and *P* < 0.05, respectively; Figure 4I,J).

**FIGURE 5:**
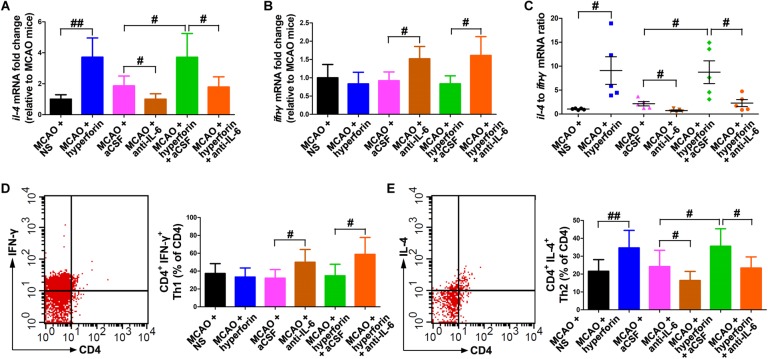
Hyperforin promoted Th2 cell differentiation in the ischemic hemisphere via IL-6 during stroke recovery. Real-time PCR analysis of the mRNA levels of *il-4* **(A)** and *ifn-γ* **(B)**, and their ratio **(C)** in the ischemic hemisphere for each group at 28 dpi. Data represent mean ± SD, *n* = 5; ^#^*P* < 0.05 and ^##^*P* < 0.01. T lymphocyte subsets of ischemic hemisphere in each group as measured by flow cytometry. The mononuclear cells were harvested from ischemic hemisphere and strained with various combinations of antibodies [Th1: CD4^+^ IFN-γ^+^ **(D)**; Th2: CD4^+^ IL-4^+^ **(E)**] at 28 dpi. Data represent mean ± SD, *n* = 5; ^#^*P* < 0.05; ^##^*P* < 0.01. aCSF, artificial cerebrospinal fluid; Th1, T helper (Th) type 1 cells; Th2, T helper (Th) type 2 cells.

To determine whether hyperforin could modulate Th1/Th2 phenotype bias, we measured mRNA expression of the cytokines *ifn-γ* and *il-4* in the ischemic hemisphere, respectively representing the Th1 and Th2 cell phenotypes. Preferential up-regulation of *il-4* expression and a down-regulation of *ifn-γ* expression was found in the ischemic hemisphere at 28 dpi with the treatment of hyperforin (all *P* < 0.05; Figure 5A,C). Blockade of IL-6 significantly decreased the mRNA expression of *il-4* and increased the mRNA expression of *ifn-γ* (all *P* < 0.05; Figure 5A–C). Anti-IL-6 led to a significant decrease in IL-4: IFN-γ ratio in the ischemic hemisphere of hyperforin-treated mice at 28 dpi (*P* < 0.05; Figure 5A–C). Furthermore, flow cytometry data showed that hyeprforin had no significant effects on Th1 (CD4^+^ IFN-γ^+^) cell differentiation (*P* > 0.05; Figure 5D), whereas significantly increased Th2 (CD4^+^ IL-4^+^) cell differentiation (*P* < 0.05) in the ischemic hemisphere at 28 dpi (Figure 5E). However, blockade of IL-6 significantly increased Th1 cell differentiation (*P* < 0.05) and decreased Th2 cell differentiation (*P* < 0.05) at 28 dpi (Figure 5D,E). Furthermore, anti-IL-6 significantly increased Th1 cell differentiation (*P* < 0.05), where decreased Th2 cell differentiation (*P* < 0.05) in the ischemic hemisphere of hyperforin-treated mice at 28 dpi (Figure 5D,E).

### Hyperforin Promoted Neuroangiogenesis in a Tregs-Dependent Manner During Stroke Recovery

**FIGURE 6:**
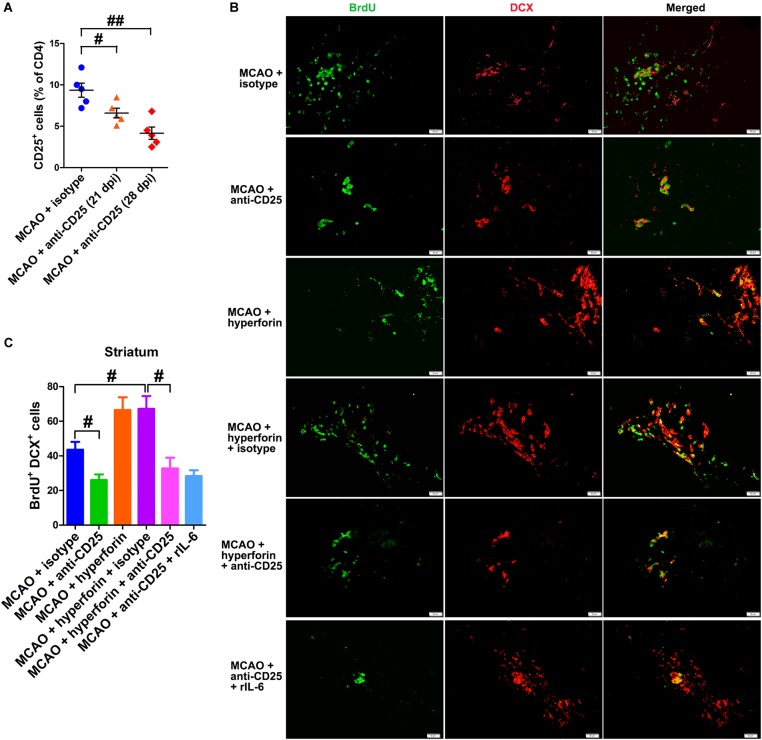
Hyperforin promoted neuroangiogenesis in a Tregs-dependent manner after ischemic stroke. **(A)** Flow cytometry analysis of cells from ischemic hemispheres after treatment with hyperforin plus anti-CD25 or isotype at 21 and 28 dpi. Data represent mean ± SD, *n* = 12; ^#^*P* < 0.05; ^##^*P* < 0.01. Double immunofluorescence labeling **(B)** and quantitative analyses **(C)** for BrdU (green) and DCX (red) for each group in the ischemic SVZ and striatum at 28 dpi. Bar = 50 μm. Data represent mean ± SD, *n* = 5; ^#^*P* < 0.05.

To investigate the effects of Tregs in the ichemic brain on the promoting effects of hyperforin on post-stroke neuroangiogenesis, CD25^+^ Tregs depleting antibody was injected at 14 and 21 dpi. With flow cytometry, CD25^+^ Tregs were detected being reduced in the ischemic hemisphere by 29% at 21 dpi and 55% at 28 dpi, compared to the isotype-treated control group at 21 dpi (*P* < 0.05 and *P* < 0.01, respectively; Figure 6A). Blockade of CD25 significantly decreased the number of BrdU^+^/DCX^+^ cells in the ischemic striatum at 28 dpi (44 ± 5 vs. 26 ± 3, *P* < 0.05; Figure 6B,C). Moreover, anti-CD25 significantly decreased the number of BrdU^+^/DCX^+^ cells in the ischemic striatum of hyperforin-treated mice at 28 dpi (67 ± 7 vs. 33 ± 6, *P* < 0.05; Figure 6B,C).

To elucidate the effects of IL-6 on Treg-dependent angioneurogenesis, rIL-6 was administrated to CD25-treated mice every 24 h for 7 days starting at 21 dpi. We found that rIL-6 treatment had no significant effects on the number of BrdU^+^/DCX^+^ cells in the ischemic striatum of CD25-treated mice at 28 dpi (*P* > 0.05; Figure 6B,C), indicating an essential role of Tregs in IL-6-mediated post-stroke angioneurogenesis.

### IL-4 and TGF-β Were Critical to the Promoting Roles of Hyperforin in Post-stroke Neuroangiogenesis and Functional Recovery

**FIGURE 7:**
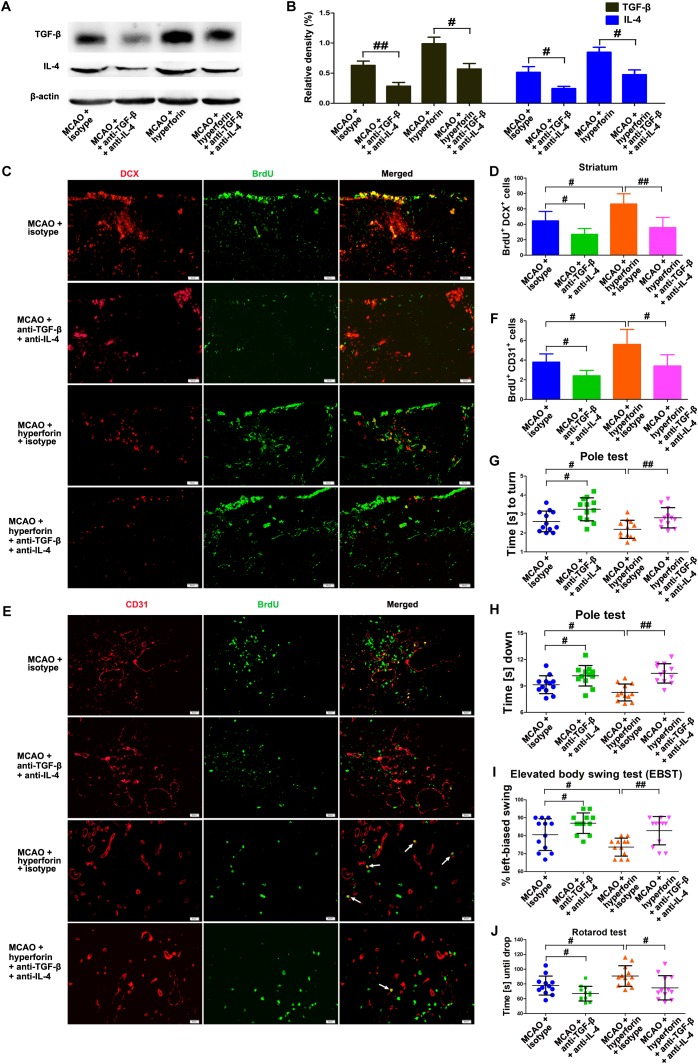
IL-4 and TGF-β are essential to the promoting effects of hyperforin on post-stroke neuroangiogenesis and functional recovery. Western blotting images **(A)** and quantitative data **(B)** for IL-4 and TGF-β in the ischemic hemisphere of each group at 28 dpi. Data represent mean ± SD, *n* = 5; ^#^*P* < 0.05, ^##^*P* < 0.01. **(C,D)** Representative images and quantitative comparison of cells double-labeled for BrdU (green) and DCX (red) for each group in the ischemic SVZ and striatum at 28 dpi. Bar = 50 μm. Data represent mean ± SD, *n* = 5; ^#^*P* < 0.05; ^##^*P* < 0.01. **(E,F)** Double immunofluorescence labeling and quantitative analyses for BrdU (green) and CD31 (red) for each group in the ischemic penumbra at 28 dpi. Bar = 20 μm. Data represent mean ± SD, *n* = 5; ^#^*P* < 0.05; ^##^*P* < 0.01. Behavioral tests were assessed at 28 dpi, including the pole test [time to turn completely head down **(G)** and time to reach the floor **(H)**], elevated body swing test (EBST) **(I)** and rotarod test **(J)**. Data represent mean ± SD, *n* = 12; ^#^*P* < 0.05; ^##^*P* < 0.01.

To determine whether Tregs and Th2 cells-mediated immunosuppressed microenvironment by secreting TGF-β and IL-4 in the ischemic hemisphere is directly involved in hyperforin-mediated promotion of neuroangiogenesis and functional recovery after ischemic stroke, blockade of IL-4 and TGF-β1 with neutralizing antibodies was performed. We found that neutralizing IL-4 plus TGF-β1 treatment effectively decreased the protein expression of IL-4 and TGF-β1 in the hemisphere (*P* < 0.05 or *P* < 0.05; Figure 7A,B). Blockade of IL-4 plus TGF-β1 significantly decreased the number of BrdU^+^/DCX^+^ cells in the ischemic striatum (45 ± 5 vs. 27 ± 3, *P* < 0.05; Figure 7C,D) and BrdU^+^/CD31^+^ cells in the ischemic penumbra at 28 dpi (4 ± 1 vs. 2 ± 1, *P* < 0.05; Figure 7E,F). Blockade of IL-4 plus TGF-β1 significantly decreased the number of BrdU^+^/DCX^+^ cells in the ischemic striatum (66 ± 5 vs. 36 ± 5, *P* < 0.01; Figure 7C,D) and BrdU^+^/CD31^+^ cells in the ischemic penumbra of hyperforin-treated mice at 28 dpi (6 ± 1 vs. 3 ± 1, *P* < 0.05; Figure 7E,F). Blockade of IL-4 plus TGF-β1 significantly increased the times to turn (*P* < 0.05; Figure 7G) and the time to reach the floor (*P* < 0.05; Figure 7H) on the pole test, increased the behavioral asymmetries on the EBST (*P* < 0.05; Figure 7I), and decreased the times until drop on the Rotarod test (*P* < 0.05; Figure 7J) at 28 dpi. Furthermore, the times to turn (*P* < 0.01; Figure 7G) and the time to reach the floor (*P* < 0.01; Figure 7H) on the pole test and the behavioral asymmetries on the EBST (*P* < 0.01; Figure 7I) were significantly increased, whereas the times until drop on the Rotarod test (*P* < 0.05; Figure 7J) at 28 dpi were significantly decreased in hyperforin-treated mice after blockade of IL-4 plus TGF-β1.

**FIGURE 8:**
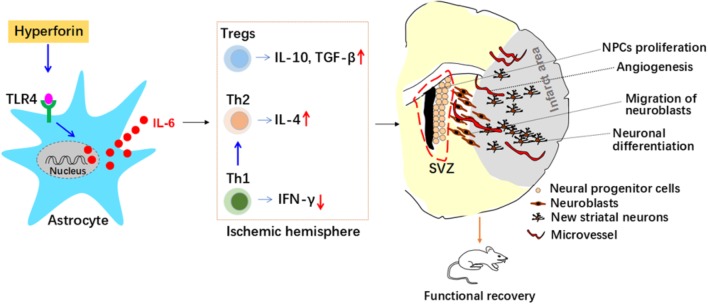
Roles and mechanisms of hyperforin in post-stroke neuroangiogenesis and functional recovery. Interleukin 6 (IL-6) secreted from astrocytes inhibits T helper (Th) 1 cell differentiation while promotes regulatory T cells (Tregs) and Th2 cell differentiation in the ischemic hemisphere, which mediated immunosuppressive environment and contributed to the promoting effects of hyperforin on neuroangiogenesis and functional recovery. Interferon-γ (IFN-γ); neural progenitor cells (NPCs); SVZ, subventricular zone; Toll-like receptor 4 (TLR4); transforming growth factor-β (TGF-β).

## Discussion

Our *in vivo* results revealed that delayed 14-days treatment with hyperforin for two continuous weeks could promote SVZ neurogenesis, angiogenesis in ischemic penumbra and thus improve functional recovery after ischemic stroke. Our further *in vivo* and *in vitro* results identified previously unrecognized mechanisms of hyperforin in ischemic stroke: astrocytic IL-6 inhibited Th1 cell differentiation while promoted Tregs and Th2 cell differentiation in the ischemic hemisphere, which mediated immunosuppressive environment and contributed to the promoting effects of hyperforin on neuroabgiogensis and functional recovery.

A strong accumulation and proliferation of Tregs in the ischemic hemisphere could be observed for up to 30 days after MCAO (Stubbe et al., 2013). However, the role of Tregs in functional recovery after ischemic stroke is controversial. The anti-CD25 antibody is injected intraperitoneally on days 3 and 14 after MCAO to effectively deplete CD25^+^ Tregs at the time a large number of CD4 lymphocyte and Foxp 3^+^ Tregs infiltration to the ischemic brain. Nevertheless, the infarct volume and neurological outcome are unchanged after the depletion of CD25^+^ Tregs at 27 days after MCAO (Stubbe et al., 2013). Furthermore, adoptive transfer of Tregs 2 h after cerebral ischemia reduces infarct volume from days 3 to 28, which is accompanied by a higher Foxp3 expression and a reduction in inflammation in the ischemic brain at day 10. However, after the adoptive transfer of Tregs in the acute phase, no changes regarding the neurogenesis and angiogenesis are detected at 29 days after MCAO (Brea et al., 2014). The findings of the above two studies indicate that the depletion of Tregs or adoptive transfer of Tregs seems to have no effects on neurological outcome or neuroangiogenesis after stroke. However, in our present study, the increase of Tregs in the ischemic hemisphere mediated by the treatment with hyperforin during stroke recovery could promote neuroangiogenesis and improve functional recovery, supporting an important role for Tregs in promoting neuroangiogenesis and recovery. Evidences have shown that boosting Tregs starting at 2 or 3 h after experimental ischemia have been shown to decrease the infarct volume at 3 and 7 days after MCAO via attenuating the inflammatory response (Brea et al., 2014; Na et al., 2015). Systemic administration of purified Tregs at 2, 6, or even 24 h after reperfusion markedly attenuates brain infarct at 3 days after MCAO via attenuating post-ischemic inflammation and reducing the early infiltration of peripheral immune cells into the brain (Li et al., 2013). Therefore, we speculate that CD25^+^ Tregs depletion-induced reduction in anti-inflammatory response in the acute phase may slightly increase the infarct volume. Previous study has provided evidence that the cell proliferation at the SVZ is dependent on infarct volume after ischemic stroke, as shown by the strong positive correlation found between infarct volume and ipsilateral SVZ cell proliferation (Moraga et al., 2014). The increase in the infarct volume in the acute phase could promote neuroangiogenesis during stroke recovery, which may be compromised by CD25^+^ Tregs depletion-mediated decrease in neuroangiogenesis. Thus, the neurological outcome were unaffected after the depletion of CD25^+^ Tregs at 27 days after MCAO (Stubbe et al., 2013). Similarly, the decrease in the infarct volume after adoptive transfer of Tregs during the acute phase induces a reduction in neuroangiogenesis during stroke recovery. However, adoptive transfer of Tregs to increase Tregs in the ischemic brain during stroke recovery may restore the reduced neuroangiogenesis mediated by smaller infarct volume in the acute phase. Thus, no changes regarding the neuroangiogenesis were detected at 29 days after MCAO after Tregs treatment in the acute phase (Brea et al., 2014).

Development of a Th1 response to MBP could lead to worse neurological outcome, whereas induction of a Th2 response could result in a better outcome for up to 1 month after MCAO (Becker et al., 2005; Gee et al., 2007). IFN-γ was used to indicate a Th1 response and IL-4, to indicate a Th2 response. Our present data found that hyperforin treatment during stroke recovery skewed differentiation of naive T cells into Th2 cells, whereas had no significant effects on Th1 cell differentiation. The possible reason may be that there was little evidence that animals developed a Th1 response to MBP up to 1 month after MCAO; on the contrary, the predominant response at 1 month was that of tolerance, developing a Th2/Th3 response to MBP (Gee et al., 2007). In our present study, examining the balance of local cytokine production in the ischemic hemisphere revealed a significant increase in the ratio of IL-4/IFN-γ after the treatment with hyperforin during stroke recovery, indicating that hyperforin could shift the balance of cytokine production from Th1 to Th2, which might add to the immunosuppressive environment of the neuroangiogenesis.

One previous study demonstrates that IL-6 promotes post-stroke angiogenesis and functional recovery (Gertz et al., 2012), but the cellular source of IL-6 during stroke recovery is unknown. Our previous studies found that astrocytes-derived IL-6 is essential to post-stroke angiogenesis and neurogenesis mediated by enriched environment and social support (Meng et al., 2015; Chen et al., 2017). In the present study, we further confirmed the promoting role of astrocytes-derived IL-6 in post-stroke neuroangiogenesis and functional recovery. We also found that hyperforin could promote neuroangiogenesis and functional recovery via astrocytic IL-6. Evidence has shown that hyperforin could inhibit Th1 cell differentiation while promoting Tregs and Th2 cell differentiation, and ultimately attenuate the severity of the CNS autoimmune disease (Nosratabadi et al., 2016). Based on the results from this study (Nosratabadi et al., 2016) combined with our results showing that astrocytic IL-6 is critical to hyperforin-mediated promoting effects on post-stroke neuroangiogenesis, we investigated the effects of IL-6 on the regulation of Th1/Th2 and Tregs response in the ischemic hemisphere during stroke recovery. We also investigated the roles of IL-6-mediated balance of cell-mediated immunity in the promoting effects of hyperforin in post-stroke neuroangiogenesis. To our knowledge, this is the first evidence revealing that astrocytic IL-6 could skew the immune response to a Th2 bias in the ischemic hemisphere and thus contribute to post-stroke neuroangiogenesis and hyperforin-mediated promoting neuroangiogenesis effects.

However, our present study has some limitations. First, the rotarod test used in our study is not a reliable method to detect motor coordination and balance. Besides the rotarod test and pole test, it would be better if other types of behavioral methods could be used to detect motor coordination and balance. Second, 3D fluorescence confocal images of double immunofluorescence labeling of BrdU and CD31 would better demonstrate the co-localization of BrdU and CD31.

## Conclusion

Our work reveals a new mechanism for the neurorepair effect of hyperforin. Hyperforin could promote post-stroke SVZ neurogenesis, angiogenesis and functional recovery through inhibiting Th1 cell differentiation while promoting Th2/Tregs cells differentiation in the ischemic hemisphere, which is mediated by astrocytic IL-6 during stroke recovery. Our present study provides new therapeutic strategies regarding astrocytes-mediated negative regulation of immunity for the treatment of ischemic stroke. Our study also provide a theoretical basis for the potential therapeutic use of hyperforin in the treatment of ischemic stroke during stroke recovery.

## Data Availability

The raw data supporting the conclusions of this manuscript will be made available by the authors, without undue reservation, to any qualified researcher.

## Ethics Statement

This study was carried out in accordance with the National Institutes of Health (NIH) Guide for the Care and Use of Laboratory Animals (Publications No. 80-23) revised 1996. The protocol was approved by the Animal Care and Use Committee for experimental animals of Tongji Medical College (Wuhan, China).

## Author Contributions

SY and JZ conceived and designed the experiments and contributed in the data analysis. HY, YZ, and HS contributed in the stroke model, RT-qPCR, immunocytochemistry, flow cytometry, western blotting, and functional assays. YY and YS helped in the immunocytochemistry and functional assays. JZ contributed in the imaging tools and writing the manuscript. All authors approved the final manuscript.

## Conflict of Interest Statement

The authors declare that the research was conducted in the absence of any commercial or financial relationships that could be construed as a potential conflict of interest.
